# The interplay between cognitive, affective, and physical activity correlates in older adults

**DOI:** 10.3389/fspor.2026.1745889

**Published:** 2026-02-27

**Authors:** Alessandro Geraci, Giovanni Angelo Navarra, Antonella D’Amico, Carla La Rizza, Laura Di Domenico, Vincenzo Di Noto, Antonino Scardina, Garden Tabacchi, Marianna Bellafiore

**Affiliations:** 1 Department of Psychology, Educational Science and Human Movement, University of Palermo, Palermo, Italy; 2 Centro Studi Internazionale MetaIntelligenze, Palermo, Italy; 3 Azienda Sanitaria Provinciale di Palermo, Palermo, Italy

**Keywords:** cognitive function, emotional intelligence, meta-emotional beliefs, older adults, physical activity, psychological well-being

## Abstract

**Background:**

The global increase in the older adult population highlights the importance of strategies to promote healthy aging, with physical activity representing one of the most effective factor. This study explored the interplay between physical activity, cognitive functioning, emotional functioning, and psychological well-being in older adults.

**Methods:**

An observational cross-sectional study was conducted with 178 autonomous older adults. Participants completed standardized assessments of physical activity (IPAQ), cognitive functioning (MMSE, CDT, Stroop, and Digit Span), emotional functioning (MSCEIT, meta-emotional beliefs, and emotional self-concept), and psychological well-being (PWBS).

**Results:**

Correlation analyses revealed positive associations between physical activity and cognitive performance, particularly working memory, as well as psychological well-being, especially personal growth. Group comparisons indicated that highly active individuals reported greater personal growth compared to inactive individuals. Regarding cognition, physical activity was positively related to working memory performance, as measured by the Digit Span Task, while no significant associations emerged with broader global cognitive screening measures such as the Mini Mental State Examination or Clock Drawing Test. In addition, physical activity was positively associated with meta-emotional beliefs, defined as individuals’ beliefs about the role of emotions in daily life, whereas no significant associations emerged with emotional abilities.

**Conclusions:**

Overall, the findings suggest that physical activity is associated with specific cognitive processes and with broader psychological resources in later adulthood. These results highlight the relevance of targeted interventions aimed at promoting physical activity to support cognitive and emotional functioning, and psychological well-being in aging populations.

## 1 Introduction

In the past five decades, the global demographic landscape has undergone significant changes due to declining birth rates and increased life expectancy. This has led to a marked shift in the age structure of populations, with a substantial increase in the proportion of older adults relative to younger cohorts (1). According to the World Health Organization (WHO), the number of people aged 60 and older is expected to double by 2050, with those aged 80 and over projected to triple between 2020 and 2050 (2). As a result, the concept of healthy ageing has gained increasing attention, emphasizing the need for policies and interventions aimed at improving the quality of life and functional independence of older adults (3–5).

Regular physical activity (PA) is one of the most effective strategies for promoting healthy ageing, offering well-established benefits for both physical and mental health. Engaging in regular PA has been linked to reduced risk of developing non-communicable diseases—including cardiovascular diseases, type 2 diabetes, certain types of cancer, and stroke—as well as to improved mood, stress regulation, and delayed cognitive decline (6–8). Despite these benefits, global levels of PA among older adults remain insufficient and often fall short of WHO guidelines (9, 10). According to the 2020 WHO recommendations, older adults should engage in 150–300 min of moderate-intensity aerobic PA, or 75–150 min of vigorous-intensity activity per week, along with muscle-strengthening exercises on two or more days per week. Moreover, multi-component PA—combining aerobic, muscle-strengthening, and balance exercises—is strongly encouraged to enhance functional ability and reduce fall risk (11).

Beyond its somatic benefits, a growing body of evidence highlights the positive influence of PA on cognitive and affective functioning in older adults. Studies have shown that regular PA is associated with improvements in executive functions (12–16), memory (17), creative thinking (18), mental health outcomes such as stress resilience and reduced symptoms of depression and anxiety (19–21), psychological well-being (22–24), and better quality of life (25, 26). These effects are likely mediated by both neurobiological mechanisms, such as increased hippocampal volume, neurogenesis, and improved cerebral blood flow, and psychosocial factors, including enhanced self-efficacy and social engagement (27, 28). A review by Mandolesi et al. (29) confirmed that PA contributes to psychological well-being by reducing dysfunctional behaviors and increasing emotional stability, self-confidence, cognitive flexibility, and internal locus of control. These findings suggest that PA may serve as a protective factor against both normal and pathological aspects of cognitive and psychological aging, thereby promoting a more resilient aging process.

In addition to its benefits for physical and cognitive health, PA also plays a significant role in emotional functioning (30). Emotions are fundamental to human nature and serve as a driving force behind behavior (31). Emotional processes are closely related to motivation, self-regulation, and adaptive behaviors, all of which are central to sustained engagement in PA and to psychological well-being in later life (32). Consistent with this perspective, recent evidence suggests that emotion regulation and related emotional constructs may mediate the association between PA and mental health indicators (33, 34). Accordingly, emotional functioning may represent an additional factor linking PA to well-being and quality of life in older adulthood. Within the emotional domain, emotional intelligence (EI) has been proposed as a key variable for understanding individual differences in how emotions are processed and regulated. Specifically, EI as defined by Mayer and Salovey (35), refers to the ability to perceive, understand, manage, and use emotions in both personal and social contexts. Previous research has found positive correlations between EI and health behaviors such as better stress management (36) and PA (31). Conversely, negative correlations have been observed between EI and behaviors like alcohol consumption and smoking (36, 37). More broadly, meta-analytic investigation show that EI is identified as an important predictor of positive health-related outcomes (38, 39) however it is important to note that the predictive validity if EI largely depends on how it is measured. To this regard, the main competing measurement approaches for EI assessment are self-report and performance-based measures (40). Over the years, there was a growing interest in the intersection of EI, sport, and exercise (41) which has led to studies showing a positive association between EI and regular physical exercise (41–43). Nevertheless, the stronger associations are mostly found with self-report measures of EI, as evidenced by a systematic review of Laborde et al. (41), in which only two studies employed performance-based measures. More recently, within the EI domain, emotional functioning is increasingly conceptualized as a multi-level process that involves not only emotional abilities, but also reflective processes concerning emotions themselves. In line with meta-emotional intelligence (MEI) framework (44), which draws on from metacognition theory (45), effective emotional functioning involves emotional abilities, self-perceptions, and beliefs about emotions. Alongside actual emotional abilities, individuals hold beliefs about emotions that operate at a meta-level, guiding how emotions are valued, monitored, evaluated and regulated in everyday life (44). These meta-emotional beliefs refer to individuals' beliefs about the relevance, usefulness, and regulation of emotions and may play a key role in shaping emotional functioning and adaptive behavior. To this regard, recent study showed that meta-emotional beliefs are positively associated with psychological well-being among pre-adolescents (46).

Taken together, the evidence reviewed indicates that PA is associated with multiple domains of functioning in later life, including cognitive, emotional, and psychological well-being. However, previous findings also suggest that these associations may differ depending on the specific cognitive and emotional processes considered. This issue is particularly relevant in older adulthood, a life stage characterized by age-related changes across multiple domains. Based on these premises, the present study aims to examine the interplay between PA, cognitive functioning, emotional functioning, and psychological well-being in older adults. Specifically, the study examines how PA relates to distinct cognitive functions, to different components of emotional functioning, namely, emotional abilities and meta-emotional beliefs, and to different dimensions of psychological well-being.

## 2 Materials and methods

### 2.1 Participants and procedure

The sample included 178 older adults (28 men and 150 women), residing in the city of Palermo and its surrounding province, who voluntarily participated in this study. The inclusion criteria were designed to ensure that only older adults without known cognitive or neurological disorders took part in the study. In particular, the absence of cognitive and physical impairments was verified using a combination of self-reported medical history and indicators of functional independence in everyday life. Participants were recruited through the Provincial Health Authority of Palermo and were required to be autonomous in daily activities, to have no evident motor or sensory deficits that could interfere with testing, and not to be receiving treatment for psychiatric or neurodegenerative disorders. Individuals who reported previous diagnoses of dementia, Parkinson's disease, stroke, or other conditions known to affect cognition or mobility were excluded.

Sample had an average age of 70.66 years (SD = 6.14). (while education levels ranged from 4 to 23 years of education (M = 10.60, SD = 4.26). Weight ranged from 15.0 kg to 123.0 kg (M = 67.52; SD = 12.38), while height ranged from 1.40 m to 1.83 m (M = 1.61; SD = 0.07). Body Mass Index (BMI) ranged from 6.67 to 44.10 (M = 25.97; SD = 4.19).

The study was performed from November 2022 to January 2023 as part of an intervention evaluation for the Physical Activity Promotion & Domestic Accidents Prevention (PAP & DAP) Project, in collaboration with Palermo's Provincial Health Authority (ASP). This project, founded in 2021, is a multidisciplinary intervention that integrated a psychological, educational and physical activity approach for promoting health and well-being in over 60 population. This 3-year project provided theoretical and practical activities aimed at preventing the main risk factors for domestic accidents, promoting active lifestyles and improving quality of life. Specifically, training protocols were implemented to improve body balance, muscle flexibility, cardiorespiratory fitness, and muscle strength. The psychological and educational approach focused on awareness of the stereotypes and emotions associated with aging; the opportunities and resources of active aging; the perception of domestic risks and educational prevention strategies; and the motivation to adopt an active lifestyle. Recruitment took place from September to November 2022, using voluntary sign-ups via posters distributed by ASP representatives in different districts of Palermo. All participants provided written informed consent, participation was entirely voluntary, with the option to withdraw at any time. This study was reviewed and approved by the local Committee (124815-04/11/2022).

### 2.2 Measures

Participants completed a series of standardized tests and questionnaires designed to assess PA levels, cognitive functioning, emotional functioning, and psychological well-being. Paper-based questionnaires were self-administered, while digital versions were completed via a Google Form accessible on smartphones, under the supervision of the research team. The two formats (paper and digital versions) were identical in terms of item content, wording, and response scales. Mixed administration modes were adopted for practical reasons, particularly to facilitate the administration process. Motor assessments were conducted by a motor science specialist, whereas cognitive and emotional assessments were administered by a psychologist.

*The International Physical Activity Questionnaire – IPAQ* (47), Italian adaptation by Mannocci et al. (48), includes 7 items designed to measure the amount of PA performed in the previous seven days. Participants were asked to report the number of days and minutes they engaged in vigorous, moderate, and walking activities, as well as the time spent sedentary. These responses were used to calculate a score reflecting the Metabolic Equivalent of Task (MET) in minutes per week (MET min/week), which represents the energy expenditure associated with PA. One MET corresponds to the energy used while at rest. For each type of PA, a specific MET value is applied by multiplying the time spent on the activity (in minutes), the number of days, and a pre-assigned MET value. Walking is assigned a value of 3.3 METs, moderate activity 4 METs, and vigorous activity 8 METs. The final score is calculated by summing the METs for each type of activity (vigorous, moderate, or walking). Based on their total MET score, participants are categorized as inactive if the total is less than 700 METs, sufficiently active if between 700 and 2,519 METs, and active or very active if above 2,520 METs.

*The Psychological Well-Being Scale – PWBS* (49) was employed to assess participants' eudaimonic well-being. It was used the short Italian version of the scale (50) composed of 18 items scored on a 5-point Likert scale (1 = completely disagree; 5 = completely agree). A higher score indicates a higher level of well-being. The PWB scale is composed of six subscales, each evaluating a different dimension of well-being: self-acceptance, positive relationships with others, autonomy, environmental mastery, purpose in life, and personal growth. The Self-Acceptance subscale measures an individual's attitude toward themselves while the Positive Relations with Others subscale evaluates the quality of one's relationships. The Autonomy subscale gauges a person's capacity for making independent decisions, and the Environmental Mastery subscale assesses how well individuals manage and adapt to their surroundings. The Purpose in Life subscale measures a sense of having goals and meaning, while the Personal Growth subscale assesses continuous personal development.

*The Clock Drawing Test – CDT* (51, 52), Italian version by Caffarra et al. (53), is a cognitive assessment tool primarily used to screen for visuospatial and executive functioning deficits, as well as to evaluate general cognitive abilities. In the CDT, participants are asked to draw a clock, typically instructed to position the hands to a specific time (e.g., “10 past 11”) within a drawn circle, including the numbers in the correct order around the clock face. Scoring for the CDT considers the accuracy of number placement, the drawing of the clock hands, and the overall spatial organization of the clock face. Errors in any of these areas can indicate cognitive impairments associated with conditions such as dementia or other neurodegenerative disorders.

*The Mini Mental State Examination – MMSE* (54), Italian version by Measso et al. (55), is a cognitive screening composed of 30 questions that cover seven cognitive domains: temporal and spatial orientation, word registration, attention and calculation, word recall, language, and constructive praxis. Scores range from 0 to 30, with one point awarded for each correct response.

*The Stroop test – ST* (56), Italian version by Caffarra et al. (57), was administered to assess inhibitory self-control in the face of verbal interferences. The test has three sections: (1) participants read words that represent colors (e.g., blue, green, red); (2) they identify the color of dots (in blue, green, or red); and (3) they state the color of the ink (blue, green, or red) used in color words (e.g., blue, green, red). Completion times for each section were recorded, and two parameters were derived: Time (indicating the degree of cognitive interference, with longer times reflecting greater interference) and Errors (where a higher error count suggests greater difficulty in managing the interference).

*The digit Span Task – Backward – DST-Bwd* (58), Italian version by Monaco et al. (59), is a test used to measure working memory, or updating ability, which is the capacity to retain information while engaging in goal-directed cognitive tasks. In this test, participants are required to repeat a sequence of digits in reverse order, with the sequence length increasing progressively. The score is determined by the longest sequence (in terms of digits) accurately recalled.

*The Digit Span Task – Forward – DST-Fwd* (58), Italian version by Monaco et al. (59), is a test that assesses short-term memory. In this version, participants repeat a sequence of digits in forward order, with the sequence length gradually increasing. The score reflects the longest sequence of digits that the participant can correctly recall. This assessment is included in the cognitive testing to help screen out participants who may have potential short-term memory impairments.

*The Meta-Emotional Beliefs Scale – CE* (60), is a self-report that consists of 16 items rated on a five-point Likert scale, from 0 (not true) to 4 (definitely true). This scale examines individuals' beliefs regarding the perception, facilitation, understanding, and regulation of emotions. The CE score reflects the extent to which individuals believe that each aspect of emotion outlined in the EI ability-based model is significant and affects daily life. For example, it assesses beliefs such as whether sensations lead to emotions, emotions aid in thinking, emotions can blend together, or emotions can be regulated. The original version developed for adolescents was adapted for use with adults, and both exploratory and confirmatory factor analyses supported the four-branch structural model (*χ*^2^(164) = 22.2, *p* = 0.075, CFI = 0.99, TLI = 0.99, SRMR = 0.02, RMSEA = 0.03, 90% CI [0.00, 0.04]). Normative data were obtained from a sample of 963 adults (805 women and 158 men), and scores are standardized, with a mean of 100 and a standard deviation of 15.

*The Emotional Self-Concept Scale – CME* (60) is a self-report that assesses self-perceived abilities in perceiving, facilitating, understanding, and managing emotions. This scale includes 20 items, also rated on a five-point Likert scale from 0 (not true) to 4 (definitely true). The items ask respondents to assess their emotional abilities in everyday situations. The score represents how capable individuals believe they are in perceiving, using, understanding, and managing emotions in daily life. Initially developed and validated for use with pre-adolescents and adolescents, the instrument was subsequently adapted for adults. Exploratory and confirmatory factor analyses supported the four-branch structural model in the adult version (*χ*^2^(164) = 38.4, *p* < 0.001, CFI = 0.98, TLI = 0.97, SRMR = 0.02, RMSEA = 0.04, 90% CI [0.03, 0.06]). The normative dataset included 963 adults (805 women, 158 men), and scores are standardized, with a mean of 100 and a standard deviation of 15.

*The Mayer-Salovey-Caruso Emotional Intelligence Test – MSCEIT* (61), Italian validation by D'Amico and Curci (62), is an ability-based measure that evaluates emotional intelligence (EI) through maximum performance. The test assesses the four key branches of ability EI (63) and consists of 141 items organized into eight tasks, each pair corresponding to one of the four EI abilities: perceiving, facilitating, understanding, and managing emotions. The MSCEIT provides fifteen primary scores: eight subscale scores, four branch scores, two area scores (experiential and strategic), and an overall emotional intelligence quotient. Participants’ responses are scored using general consensus norms. Scores were calculated using the consensus-based scoring approach (61), which assigns higher scores to responses that align with the most frequently selected answers in the large normative sample. This approach reflects the assumption that emotional intelligence involves socially shared understandings of emotional situations, with correctness determined by the degree of agreement with the broader population. The normative sample used for consensus scoring consists of 3,185 adults (1,718 females and 1,487 males) and corresponds to the Italian standardization sample reported in the Italian validation of the MSCEIT (62). Scores are standardized, with a mean of 100 and a standard deviation of 15.

### 2.3 Data analysis

All statistical analyses were performed using IBM SPSS Statistics, version 25.0 (64). First, prior to the main analyses, assumptions underlying parametric tests were examined. Distributional properties of the variables were inspected, and assumptions of normality and homoscedasticity were evaluated. Second, descriptive statistics were calculated for all study variables and Pearson correlation coefficients were computed to examine the bivariate associations between PA levels, measured as Metabolic Equivalent of Task (MET), and psychological well-being, cognitive and emotional variables. Third, to derive a composite indicator of general cognitive functioning, a principal component analysis (PCA) was conducted on cognitive test scores. The PCA included measures of working memory, attentional and inhibitory control, and visuospatial abilities, while the MMSE was considered separately as a global screening measure. Cognitive variables were coded so that higher scores reflected better performance. Sampling adequacy was verified using the Kaiser–Meyer–Olkin measure and Bartlett's test of sphericity. A single-component solution was retained and component scores were saved for subsequent analyses examining associations with PA, emotional dimensions, and psychological well-being. Fourth, in addition to correlation analyses, participants were classified into three groups based on their level of PA (i.e., inactive, sufficiently active, and very active), according to the IPAQ scoring guidelines and total MET minutes per week. This grouping was used to examine whether different levels of PA were associated with differences in cognitive functioning, emotional dimensions, and psychological well-being. Accordingly, a series of one-way Analyses of Variance (ANOVAs) were conducted on the dependent variables related to cognitive performance (e.g., MMSE, Clock Drawing Test, Stroop Test, Digit Span Forward and Backward), emotional dimensions (e.g., Meta-Emotional Beliefs, Emotional Self-Concept, MSCEIT), and psychological well-being (e.g., the six Ryff subscales). Lastly, when statistically significant main effects were found, Bonferroni *post-hoc* tests were used to explore pairwise differences between groups. Effect sizes were reported using partial eta squared (partial *η*^2^).

## 3 Results

### 3.1 Correlational analyses among PA, cognitive, emotional and psychological dimensions

Table 1 presents descriptive statistics (i.e., mean and standard deviation) and the Pearson correlation coefficients between the study variables. The correlation matrix highlights several relationships between PA, as measured by the Metabolic Equivalent of Task (MET), and various psychological, emotional and cognitive measures.

**Table 1:** Correlation analysis results among the study variables.

Scale	1	2	3	4	5	6	7	8	9	10	11	12	13	14	15	16
1.PWBS-PR	—															
2.PWBS-A	−0.16*	—														
3.PWBS-EM	−0.02	0.45***	—													
4.PWBS-PG	0.04	0.17*	0.09	—												
5.PWBS-PL	0.14	−0.01	0.23**	0.15	—											
6.PWBS-SA	0.04	0.27***	0.29***	0.06	−0.13	—										
7.MET	0.11	0.01	−0.02	0.20*	0.13	0.09	—									
8.MMSE	−0.02	−0.00	−0.02	−0.12	0.16	−0.23*	0.03	—								
9.CDT	0.13	−0.30**	−0.11	−0.35***	−0.03	0.00	−0.10	−0.07	—							
10.ST-Time	0.13	−0.18	0.03	−0.20	0.05	0.06	−0.14	−0.05	0.08	—						
11.ST-Error	−0.01	−0.23*	0.12	−0.24*	0.22*	0.08	−0.09	−0.04	0.27**	0.33**	—					
12.DST-Fwd	0.03	0.13	0.07	0.14	0.11	−0.04	0.14	0.23*	−0.17	−0.14	−0.19*	—				
13.DST-Bwd	−0.01	0.10	0.03	0.18	−0.09	−0.06	0.30**	0.15	−0.23*	−0.10	−0.25**	0.35**	—			
14.CE	−0.23	−0.09	−0.15	0.30*	0.07	−0.28*	0.28*	0.20	−0.26*	−0.10	−0.20	0.19	0.18	—		
15.CME	−0.09	0.03	0.08	−0.01	0.18	−0.05	0.09	0.03	−0.26*	0.01	−0.05	0.08	0.13	0.42**	—	
16.MSCEIT	−0.24	0.00	0.23	0.07	0.21	−0.17	−0.15	0.40*	−0.28	−0.13	0.04	0.33	0.29	0.29	0.15	—

CDT, Clock Drawing Test; CE, MetaEmotional Beliefs; CME, Emotional Self-Concept; DST-Bwd, Digit Span Task – Backward; DST-Fwd, Digit Span Task – Forward; MET, Metabolic Equivalent of Task; MMSE, Mini Mental State Examination; MSCEIT, Mayer-Salovey-Caruso Emotional Intelligence Test; PWBS-A, Psychological Well-Being Scale – Autonomy; PWBS-EM, Psychological Well-Being Scale – Environmental Mastery; PWBS-PL, Psychological Well-Being Scale – Purpose in Life; PWBS-PG, Psychological Well-Being Scale – Personal Growth; PWBS-PR, Psychological Well-Being Scale – Positive Relations with Others; PWBS-SA, Psychological Well-Being Scale – Self-Acceptance; ST, Stroop Test.

* *p* < 0.05.

** *p* < 0.01.

*** *p* < 0.001.

A significant positive correlation was found between MET and Digit Span Task – Backward (DST-Bwd: r = 0.30, *p* < 0.01), indicating that higher PA levels are associated with better working memory capacity. However, the association between MET and Digit Span Task – Forward was not significant (DST-Fwd: r = 0.14, *p* > 0.05). Moreover, no significant association between MET and the Clock Drawing Test was found (CDT: r = −0.10, *p* > 0.05).

MET also showed significant positive relationships with emotional and psychological measures. It was positively correlated with PWBS-Personal Growth (PWBS-PG: r = 0.20, *p* < 0.05), implying that individuals with higher PA levels tend to experience a greater sense of personal growth. Additionally, MET exhibited significant positive correlations with CE (Meta-Emotional Beliefs: r = 0.28, *p* < 0.05), suggesting that higher levels of PA are associated with more positive beliefs about emotions and emotional regulation. However, MET did not show significant correlations with other measures of psychological well-being or emotional dimensions.

### 3.2 Principal component analysis of cognitive functioning and associations with PA, psychological and emotional dimensions

To obtain a composite indicator of cognitive functioning, a principal component analysis (PCA) was conducted on cognitive test scores assessing working memory, attentional control, and visuospatial abilities (Digit Span Forward and Backward, Stroop Test indices, and Clock Drawing Test). The MMSE was excluded from the PCA and treated separately as a global screening measure. All cognitive variables were coded so that higher scores reflected better cognitive performance. Sampling adequacy was acceptable (Kaiser–Meyer–Olkin measure = 0.64), and Bartlett's test of sphericity was significant, *χ*^2^(10) = 48.0, *p* < 0.001. The PCA yielded a single component explaining 37.2% of the total variance (eigenvalue = 1.86). This component showed meaningful positive loadings across all included cognitive measures and was interpreted as an index of general cognitive functioning (GCF). The loadings of each cognitive measure on the first principal component are reported in Table 2.

**Table 2:** Principal component analysis (PCA) of cognitive functioning.

Cognitive measures	PC loading
ST Error	0.694
DST-Bwd	0.660
DST-Fwd	0.614
CDT	0.568
ST Time	0.491

PCA, principal component analysis; PC, principal component (general cognitive functioning). Higher scores indicate better cognitive performance. KMO, 0.64; Bartlett's test of sphericity: *χ*^2^(10) = 48.0, *p* < 0.001. PC explained 37.2% of the total variance.

Pearson correlation analyses were conducted to examine the associations between the general cognitive functioning index (GCF), PA levels, emotional dimensions, and psychological well-being. Correlation coefficients are presented in Table 3.

**Table 3:** Pearson correlations between general cognitive functioning (GCF), physical activity, emotional functioning, and psychological well-being.

Variable	*r* with GCF
MET	0.26*
PWBS-PR	−0.05
PWBS-A	0.30**
PWBS-EM	0.02
PWBS-PG	0.37***
PWBS-PL	−0.09
PWBS-SA	−0.07
CE-TOT	0.32*
CME-TOT	0.17
MSCEIT-TOT	0.32

Only correlations involving GCF are reported to avoid redundancy with Table 1.

* *p* < 0.05.

** *p* < 0.01.

*** *p* < 0.001.

Results showed that GCF was positively associated with MET (r = 0.26, *p* < 0.05), indicating that higher levels of PA were related to better overall cognitive functioning. Moreover, significant positive associations emerged between GCF and the psychological well-being dimensions of personal growth (r = 0.37, *p* < 0.001) and autonomy (r = 0.30, *p* < 0.01), suggesting that better cognitive functioning was linked to greater perceptions of self-development and self-determination.

With respect to emotional dimensions, GCF showed a significant positive association with meta-emotional beliefs (CE: r = 0.32, *p* < 0.05). In contrast, no significant associations were observed between GCF and emotional self-concept (CME: r = 0.17, *p* > 0.05) or emotional abilities (MSCEIT: r = 0.32, *p* > 0.05).

### 3.3 Group differences in cognitive, psychological, and emotional dimensions by PA levels

Table 4 presents the results of one-way ANOVA tests comparing the psychological well-being and cognitive measures in inactive (25.3%), sufficiently active (32.9%) and very active (41.8%) groups. *post hoc* pairwise comparisons were conducted using Bonferroni correction to control for multiple comparisons.

**Table 4:** Descriptive statistics and ANOVAs results for physical activity levels.

Variable	Inactive (A)			Sufficiently active (B)			Very active (C)			F_(df)_	*p*-value	Partial *η*^2^	Bonferroni *post-hoc* tests
	M	SD	N	M	SD	N	M	SD	N				
PWBS-PR	8.49	1.50	39	8.35	1.56	49	8.39	1.86	62	0.08 (_2,147)_	0.924	0.00	–
PWBS-A	10.72	2.09	39	11.35	2.09	49	11.08	2.21	62	0.94 (_2,147)_	0.394	0.01	–
PWBS-EM	9.41	1.62	39	10.10	2.00	49	9.82	2.21	62	1.30 (_2,147)_	0.276	0.02	–
PWBS-PG	10.92	1.91	39	11.27	1.62	49	11.84	1.88	62	3.33*(_2,147)_	0.039	0.04	C > A*
PWBS-PL	8.03	2.37	39	8.63	2.21	49	8.74	2.40	62	1.22 (_2,147)_	0.299	0.02	–
PWBS-SA	11.77	2.36	39	11.71	2.14	49	12.18	2.03	62	0.76 (_2,147)_	0.469	0.01	–
MET	233.63	225.21	40	1,371.39	503.98	52	5,497.29	3,937.70	66	64.39***(_2,155)_	0.000	0.45	C > A, B***
MMSE	26.23	2.09	24	26.87	2.09	34	26.80	1.84	41	0.84 (_2,38)_	0.435	0.02	–
CDT	2.79	1.67	24	2.44	1.64	34	2.05	1.22	40	1.93 (_2,95)_	0.151	0.04	–
ST-Time	32.58	20.08	24	26.61	15.36	34	28.69	14.08	38	0.96 (_2,93)_	0.386	0.02	–
ST-Error	1.92	2.65	24	1.35	2.39	34	1.63	3.29	38	0.28 (_2,93)_	0.756	0.01	–
DST-Fwd	5.12	1.12	24	5.53	0.93	34	5.51	1.00	39	1.40 (_2,94)_	0.251	0.03	–
DST-Bwd	3.21	0.98	24	3.03	0.97	34	3.85	1.20	39	5.79**(_2,94)_	0.004	0.11	C > B**
CE	97.23	11.69	13	101.48	13.25	27	103.23	11.99	31	1.06 (_2,68)_	0.351	0.03	–
CME	96.31	11.29	13	96.67	12.96	27	100.06	10.14	31	0.82 (_2,68)_	0.444	0.02	–
MSCEIT	100.60	15.81	5	104.73	14.84	15	92.52	15.18	21	2.94 (_2,38)_	0.065	0.13	–

* *p* < 0.05.

** *p* < 0.01.

*** *p* < 0.001.

Significant group differences were found in the Metabolic Equivalent of Task (MET) [F(2, 155) = 64.39, *p* < 0.001, partial *η*^2^ = 0.45], with very active individuals (M = 5,497.29, SD = 3,937.70) showing significantly higher MET scores compared to sufficiently active (M = 1,371.39, SD = 503.98) and inactive individuals (M = 233.63, SD = 225.21). Additionally, significant differences were observed in Backward Digit Span Task (DST-Bwd) [F(2, 94) = 5.79, *p* = 0.004, partial *η*^2^ = 0.11], with very active individuals (M = 3.85, SD = 1.20) outperforming sufficiently active individuals (M = 3.03, SD = 0.97).

Significant differences emerged for PWBS-Personal Growth [F(2, 147) = 3.33, *p* = 0.039, partial *η*^2^ = 0.04], with *post-hoc* Bonferroni comparisons revealing that very active individuals (M = 11.84, SD = 1.88) reported significantly higher personal growth compared to inactive individuals (M = 10.92, SD = 1.91). No significant differences were found between groups on other PWBS subscales, including Positive Relations [F(2, 147) = 0.008, *p* = 0.924], Autonomy [F(2, 147) = 0.094, *p* = 0.394], Environmental Mastery [F(2, 147) = 1.30, *p* = 0.276], Purpose in Life [F(2, 147) = 1.22, *p* = 0.299], and Self-Acceptance [F(2, 147) = 0.076, *p* = 0.469].

No significant differences were found between PA groups for other cognitive measures, including MMSE [F(2, 38) = 0.84, *p* = 0.435], Clock Drawing Test [F(2, 95) = 1.93, *p* = 0.151], Stroop Test Time [F(2, 93) = 0.96, *p* = 0.386], Stroop Test Errors [F(2, 93) = 0.28, *p* = 0.756], Forward Digit Span Task (DST-Fwd) [F(2, 94) = 1.40, *p* = 0.251]. Additionally, no significant differences were found between levels of PA and Meta-Emotional Beliefs (CE) [F(2, 68) = 1.06, *p* = 0.351], Emotional Self-Concept (CME) [F(2, 68) = 0.82, *p* = 0.444], and Mayer-Salovey-Caruso Emotional Intelligence Test (MSCEIT) [F(2, 38) = 2.94, *p* = 0.065].

## 4 Discussion

This study aimed to explore the interplay between PA, cognitive functioning, psychological and emotional well-being in older adults. The reported gender asymmetry reflects the characteristics of the reference population and the recruitment method, which was voluntary. Older women are most likely to have a higher perceived risk of falling than their male counterparts. Indeed, research showed that older women are more prone to falls than men (65). This phenomenon might be linked to greater longevity, lower muscle mass and bone density, and a higher incidence of chronic diseases (66). While not invalidating the internal validity of the analyses, this gender distribution suggests caution in extending the results to populations with a different demographic composition.

Our findings suggest significant associations between PA and both cognitive and emotional aspects of well-being, with implications for the promotion of PA as a means to enhance mental and emotional health in older populations.

Consistent with the first set of results, the present findings indicate that higher levels of physical activity are associated with better cognitive functioning in older adults, particularly with working memory. This supports existing literature suggesting that PA can improve cognitive functions, particularly executive functions (12, 13, 15, 16). Working memory plays a crucial role in everyday functioning, as it supports reasoning, problem-solving, and the ability to follow conversations or manage daily tasks (67, 68). Its preservation is therefore particularly relevant for maintaining autonomy and quality of life in older adults (68, 69). Interestingly, PA did not show significant correlations with other cognitive measures such as the Clock Drawing Test (CDT) and the Mini Mental State Examination (MMSE). The MMSE is a global cognitive screening tool that primarily captures deficits related to memory, orientation, attention, and language (54), while the CDT assesses visuospatial abilities, executive function, and planning (51, 52). Results suggest that the cognitive benefits of PA in this cohort might be more specific to working memory rather than general cognitive decline or visuospatial abilities. This aligns with research indicating that PA can benefit certain cognitive domains while not necessarily protecting against broader cognitive impairments (14, 16). Beyond domain-specific cognitive measures, the present study also examined general cognitive functioning using a composite index derived from principal component analysis. This approach allowed us to capture shared variance across multiple cognitive domains, including working memory, inhibitory control, and visuospatial abilities. Importantly, the positive association observed between PA and the general cognitive functioning index suggests that higher levels of PA are also linked to better overall cognitive functioning.

Turning to the second set of results, PA also demonstrated significant positive correlations with psychological well-being especially the dimension of personal growth suggesting that more physically active individuals report higher levels of personal development and fulfilment. This is consistent with previous studies indicating that PA enhances psychological well-being and vice versa (22, 23). Group comparisons based on PA levels revealed that individuals who were very active reported significantly higher levels of personal growth compared to inactive individuals. This finding further supports the notion that PA may be particularly beneficial for personal development in older adults (25, 26). A recent study showed that as personal growth increased, fear of falling decreased in a sample of independent elderly subjects, contributing to a better quality of life (24).

Notably, although other well-being dimensions did not differ significantly between activity levels, the link between PA and personal growth underscores the role of PA in fostering self-realization among older adults. In a previous study, SEM analysis showed that functional fitness strongly predicted both perceived physical and mental health in older adults, while PA level was associated only with the physical health component. These findings highlight the importance of promoting PA to enhance fitness and perceived health in later life (1). In this context, the positive associations observed between general cognitive functioning, personal growth, and autonomy suggest a close and reciprocal interplay between cognitive resources and eudaimonic aspects of well-being, such as self-development and self-determination. These findings are consistent with lifespan perspectives emphasizing the mutual interplay between cognitive functioning and psychological well-being in later adulthood (70, 71).

Lastly, referring to emotional functioning, the results show a differentiated pattern depending on the specific emotional components considered. With regard to EI, previous research on the association between EI and sport or PA has been increasing and often reports positive associations (41). However, inconsistencies remain largely due to the variety of competing EI measures and differing conceptualizations of how EI relates to PA. In the present study, no significant differences were found in EI across activity level groups, aligning with previous findings that report weak or non-significant associations between PA and EI (72, 73). One potential explanation for the lack of significant effects lies in the nature of the MSCEIT tool, which assesses EI as a set of abilities rather than self-perceived emotional competencies. Unlike self-report measures, which may be more sensitive to social desirability and self-presentation biases (74, 75), the MSCEIT aims to capture actual emotional problem-solving abilities. These abilities are considered relatively stable over time and may require long-term, targeted training in order to be meaningfully improved (76).

On the contrary, meta-emotional beliefs, conceptualized as individuals' beliefs about the role, usefulness, and significance of emotions in daily life, were positively associated with levels of PA. This finding suggests that reflective components of emotional processing may be related to engagement in PA. Consistently, meta-emotional beliefs were also positively associated with dimensions of psychological well-being, both in the present sample of older adults and in previous studies conducted with pre-adolescents (46). Meta-emotional beliefs thus represent an important reflective component of emotional functioning that shapes how individuals interpret and integrate emotional experiences into everyday behavior. Such beliefs may foster greater awareness of internal states and promote adaptive self-regulation, thereby supporting both psychological well-being and engagement in health-promoting behaviors, such as regular PA.

### 4.1 Limitations and future directions

While the findings of this study provide valuable insights into the relationship between PA, cognitive function, and emotional well-being in older adults, several limitations should be considered when interpreting the results. While significant correlations were observed between PA and cognitive/emotional outcomes, it remains unclear whether PA directly influences cognitive function and emotional well-being, or if other factors contribute to these relationships. Longitudinal studies are needed to establish causal pathways and better understand the long-term effects of PA on cognitive and emotional health. PA was assessed using IPAQ, which relies on self-reported data. Self-report measures are susceptible to biases such as overreporting or underreporting, as participants may inaccurately recall or exaggerate their activity levels. Objective measures of PA, such as accelerometers or pedometers, could provide more accurate data and strengthen the validity of the findings. The cognitive and emotional assessments used in this study may not fully capture all aspects of cognitive functioning and emotional well-being. Future studies could benefit from using a broader range of cognitive and emotional assessments to capture a more comprehensive view of these domains. It is also important to note that self-report measures were administered using both paper-and-pencil and digital formats. Although the two formats were identical in terms of item content, wording, and response scales, the use of mixed administration modes may represent a potential source of measurement variability. The study sample consisted predominantly of women which may limit the generalizability of the findings to men. Future studies should aim for a more balanced gender representation.

## 5 Conclusions

The findings of this study have important implications for interventions aimed at promoting PA among older adults. Encouraging older individuals to engage in regular PA may not only improve physical health but also be associated with better cognitive and emotional functioning. Beyond domain-specific findings, the use of a composite indicator of general cognitive functioning highlighted that higher levels of PA are linked to better overall cognitive functioning, suggesting a broad cognitive profile rather than isolated cognitive advantages.

Moreover, general cognitive functioning was positively associated with eudaimonic dimensions of psychological well-being, particularly personal growth and autonomy, as well as with meta-emotional beliefs. These findings point to a close interplay between cognitive resources, reflective beliefs about emotions, and psychological well-being, supporting a multidimensional perspective on active and healthy aging.

Overall, these results emphasize the relevance of targeted interventions that incorporate PA as a key component in promoting cognitive functioning psychological and emotional well-being in aging populations. Although the cross-sectional design does not allow causal inferences, the present study underscores the potential role of PA as an important modifiable factor within a broader network of cognitive, emotional, and psychological processes contributing to quality of life in later adulthood.

## Data Availability

The raw data supporting the conclusions of this article will be made available by the authors, without undue reservation.
